# The Mediating Role of Self-Exertion on the Effects of Effort on Learning Virtues and Emotional Distress in Academic Failure in a Confucian Context

**DOI:** 10.3389/fpsyg.2016.02047

**Published:** 2017-01-09

**Authors:** Bih-Jen Fwu, Shun-Wen Chen, Chih-Fen Wei, Hsiou-Huai Wang

**Affiliations:** ^1^Center for Teacher Education, National Taiwan UniversityTaipei, Taiwan; ^2^Institute of Learning Sciences, National Tsing Hua UniversityHsinchu, Taiwan; ^3^Department of Psychology and Counseling, University of TaipeiTaipei, Taiwan

**Keywords:** self-exertion, effort, learning virtues, emotional distress, Confucianism

## Abstract

Previous studies have found that in East Asian Confucian societies, hardworking students are often trapped in a dilemma of enjoying a positive moral image while suffering from emotional distress due to academic failure. This study intends to further explore whether the cultural-specific belief in self-exertion acts as a psychological mechanism to lessen these students’ negative emotions. A group of 288 college students in Taiwan were administered a questionnaire to record their responses to past academic failures. The results from structural equation modeling showed that self-exertion functioned as a mediator between the effects of effort on learning virtues and emotional distress. Self-exertion to fulfill one’s duty to oneself positively mediated the effect of effort on learning virtues, whereas self-exertion to fulfill one’s duty to one’s parents negatively mediated the effect of effort on emotional distress. Theoretical and cultural implications are further discussed.

## Introduction

Because East Asian societies in the Confucian circle place much emphasis on effort in academic achievement (Hau and Ho, 2010), students are under great pressure from their parents, teachers, and peers to make effort to pursue academic success (Ang and Huan, 2006; Tan and Yates, 2011). Given the high value of academic achievement in these societies, academic failure tends to be a serious blow to students and cause severe emotional distress. Previous studies have found that effortful Asian students tend to be trapped in a dilemma of enjoying a positive moral image while suffering from emotional distress from academic failure (Fwu et al., 2016b). Despite the positive image, they are often distraught with negative emotions, including loss of face, shame, guilt, anxiety, and depression (Matsumoto, 1991; Mortenson, 2006; Lei, 2009; Tao and Hong, 2014). It is important to inquire if there are any cultural-specific mechanisms that may help these students lessen distressed feelings and cope with academic failure.

Hau and Ho (2010) reviewed evidence on Asian students’ motivation for academic achievement and found inconsistency in applying Western achievement motivation theories, such as intrinsic vs. extrinsic motivation (Iyengar and Lepper, 1999; d’Ailly, 2003), self-efficacy and competence beliefs (Leung, 2002; Salili et al., 2004), or salience of mastery vs. performance goal (Ho et al., 2007; Ho and Hau, 2008), to East Asian contexts. The roots to such inconsistency may be traced back to the fundamental views about learning. In the Western intellectual tradition, the purpose of learning is to understand the external world, and its process focuses on developing cognitive/intellectual capacity (Li, 2012). On the other hand, in the East Asian cultures, the purpose of learning is to cultivate internal virtues, and its process focuses on perfecting oneself, with an emphasis on diligence and persistence (Li, 2012).

Central to these East Asian views about learning for self-perfection is the cultivation of one’s virtues and fulfillment of filial obligations to one’s parents (Li, 2002, 2005, 2012). On one hand, an individual needs to improve oneself constantly to possess learning virtues such as diligence, perseverance, and earnestness. On the other hand, a person has to fulfill filial obligations to glorify his or her parents by attaining high level of achievement. Therefore, making the upmost effort to fulfill (self-exertion, *jin-ji*) the dual obligation toward self and parents may serve as both a criterion to evaluate one’s learning virtues and a buffer to alleviate negative emotions resulting from failure.

Thus, the present study aims to investigate the role of self-exertion in the relationship among effort, learning virtues and emotional distress. More specifically, we wonder if effort positively predicts self-exertion; second, if effort positively predicts learning virtues and emotional distress; and third, if self-exertion positively predicts learning virtues but negatively predicts emotional distress. In the following sections, specific hypotheses were further developed based on theoretical inferences.

### Vertical Achievement Goal in Confucian Cultural Context

According to Chen et al. (2009), there are two types of achievement goal, personal vs. vertical goals, in the Confucian cultural context. A *personal goal* is defined as a goal constructed on the basis of autonomous interest and self-determined choice. Children are given the freedom to pursue or give up such a goal. The pattern of learning motivation and behaviors based on personal goals is rather universal and can be explained by the existing achievement motivation theories. On the other hand, a *vertical goal*, which is based on social expectations, is shaped by cultural and social values. Individuals’ performances in the pursuit of these goals will be ranked on a vertical ladder of achievement by others. Individuals are usually under great pressure to compete with their peers to excel and have little choice but to climb higher and higher up the “achievement pyramid” by getting good grades, going to top schools, getting high-paying jobs, acquiring high status and fame, and so on. Individuals are obligated to fulfill their role as filial children to pursue such goals to satisfy parental expectations (Tseng, 2004; Chen et al., 2009; Hwang, 2012; Huang et al., 2015; Fwu et al., 2016a,b). Those who fail to pursue vertical goals tend to feel that they have failed their obligation to their parents. In many East Asian countries, academic achievement is often viewed as a vertical goal for students (King and McInerney, 2014; Tao and Hong, 2014; Fwu et al., 2016a,b). Empirical studies have shown that Taiwanese students perceive a significant difference between academic (vertical) and non-academic (personal) goals. For them, the pursuit of academic achievement is characterized by a stronger sense of role obligation, higher parental expectations, greater social importance, and less personal choice than non-academic pursuits such as hobbies and sports (Fwu et al., 2016a,b). Major achievement motivation theories developed in the West may have overlooked such the culturally unique characteristics of vertical goals, and this may be the reason why the learning performances and behaviors of some East Asian students could not be fully understood.

As vertical goals are so crucial in this cultural context, adults tend to instill the value of vertical goals into the minds of youngsters through the process of socialization at home and in school. In the process, adults’ goals for pursuing vertical achievement are so embedded in the consciousness of young children that such goals may be gradually experienced as the children’s own goals (Markus and Kitayama, 1991). Children learn to adopt the values and beliefs of their parents about the vertical goals and gradually integrate the goals into their own pursuits (Chao, 1994, 1996). In this way, vertical goals become a common pursuit shared by both parents and children. Through realizing such consensually shared goals, children tend to incorporate and adjust themselves to fulfilling their role obligations in order to maintain harmonious relationships with their parents (Triandis, 1989; Markus and Kitayama, 1991; Su et al., 1999; Heine et al., 2001).

### Two Types of Self-Exertion in the Pursuit of Vertical Achievement Goals

In Confucian thought, self-exertion is the fundamental principle of being a moral person. According to Confucius’ Analects, his doctrine can be summarized in one phrase: exerting yourself to the utmost in order to perfect yourself in both the moral and the social realms. On the one hand, individuals should try their best continuously to cultivate virtues and character. On the other hand, they should also do their best to maintain ethical and harmonious relationships with their parents. In this way, self-exertion *(jin ji*) in the Confucian tradition is defined as “exerting the utmost effort” to fulfill the dual obligation to oneself morally and to one’s parents socially in pursuit of vertical goals.

Underlying the obligation for parents is the deep-rooted Confucian ethical principle for ordinary people (Hwang, 1999, 2012). Since individuals’ lives are the continuation of their parents’ physical lives, signifying an inseparable blood bond between parents and children, mutual fulfillment of moral obligations is prescribed: parents should be benevolent, and children should be filial (*fu ci zi xiao*) (Hwang, 1999, 2012; De Bary, 2003; Fwu et al., 2014). Children’s education is considered an arena for reciprocal obligation. Benevolent parents should fulfill their duty by providing their children with the best education possible; in return, filial children should do their duty and study hard (Hwang, 1999, 2012).

Moreover, the root to fulfilling one’s obligation to the self derives from the need to realize one’s optimal potential through a continuous process of self-perfection. In Confucianism, it is widely believed that an individual’s talents and attributes are endowed by Heaven. Only through exerting the utmost effort to do what one is obligated to do can one’s potential be realized to the ultimate level, and then one’s purpose/destiny of life, which is decreed by Heaven, can be revealed (Hwang, 2012). In this way, an individual has fulfilled his duty to his own life (*jinxi zhixing yi zhi tien*). In the case of pursuing the consensually shared goal of academic achievement, an individual can exert himself to self-perfect in order to realize his full potential, while simultaneously fulfilling this filial obligation to parents. Furthermore, such self-exertion to pursue academic achievement also signifies a character building process. By exerting the utmost effort in academic learning, an individual gradually cultivates such virtuous qualities as concentration, earnestness, diligence, persistence, and endurance of hardship (Li, 2012). Therefore, self-exertion to fulfill one’s duty to both one’s parents and oneself are closely related because oftentimes fulfilling one’s filial duty is a way to accomplish one’s personal duty, based on the inseparable relationship between parents and children.

### Self-Exertion as a Mediator between Effort and Virtue vs. Distress

Since cultural beliefs, meanings, and values encourage corresponding psychological processes, individuals living in a certain cultural context tend to absorb salient cultural beliefs and develop conforming psychological tendencies (Kitayama and Markus, 1999). Individuals who are brought up in a Confucian cultural system stressing the moral value of self-exertion tend to develop a psychological disposition to appraise the appropriateness of their conduct and regulate their emotions in order to adhere to the cultural norms of self-exertion (Hwang, 2013). According to the teachings of the Confucian classics on self-exertion (*jin-ji*), if an individual fails to achieve his goals, he should not attribute the failure to external factors or blame others; instead, he should reflect on whether he has exerted the utmost effort in the process (*xin you bu de, fan qiu zhu ji*) (Dobson, 1963). If an individual decides he has tried his best, he is assured of having fulfilled his obligations both to self and to parents. Therefore, the degree of effort is the criterion by which one evaluates whether one has exerted oneself, for both one’s parents and oneself. The more effort one has put in, the more effort one has exerted. We hypothesize that effort positively predicts exertion to oneself (H1) and to parents (H2).

Furthermore, effort also has an impact on learning virtues and negative emotion. Previous studies indicated that the more effort one expends, the more virtuous qualities, such as diligence and responsibility, one may possess (Fwu et al., 2014, 2016a,b). We hypothesize that effort positively predicts learning virtues (H3). Previous research has also indicated that the more effort one expends, the more emotional distress one may suffer (Fwu et al., 2016b). The emotional distress may result from a lack of competence and violation of the “just world” belief that one should reap what one sows. We hypothesize that effort positively predicts emotional distress (H4).

**FIGURE 1:**
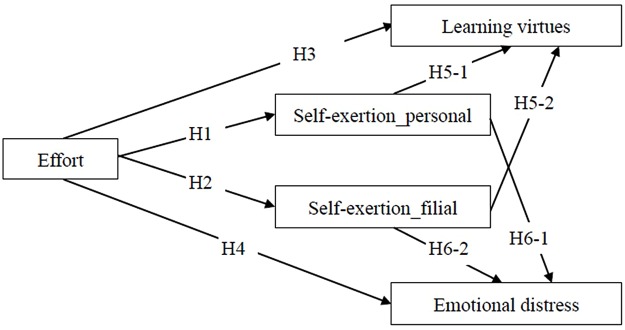
**Hypothetical framework**.

On top of effort, self-exertion also plays a role in predicting learning virtues and emotional distress. There are two types of self-exertion: one to the self (self-exertion_personal, SE-P), and the other to one’s parents (self-exertion_filial, SE-F). The degrees of both SE-P and SE-F are the criteria for evaluating the extent of one’s possession of learning virtues. The more SE-P and SE-F one has expended, the more learning virtues one possesses. We hypothesize that SE-P and SE-F positively predict learning virtues (H5-1 and H5-2). Lastly, self-exertion also functions as a regulatory factor to reduce emotional distress. After reflecting on failure, if an individual is assured that he has exerted himself in the process, he will not feel indebtedness, either to himself or to others. Such peace of mind and a clear conscience would help to regulate the negative emotions. The more SE-P and SE-F one has expended, the less distress one may experience. We hypothesize that SE-P and SE-F negatively predict emotional distress (H6-1 and H6-2). In summary, the proposed model illustrated in **Figure 1** demonstrates the hypothetical framework integrating the above hypotheses in this study.

In sum, the aim of the study is to inquire the cultural-specific mechanisms that may help East Asian students lessen distressed feelings and cope with academic failure. More specifically, we intend to examine the role of self-exertion *(jin-ji)* in the relationship among effort, learning virtues and emotional distress. We hypothesize that effort positively predicts self-exertion to self and to parents; effort positively predicts learning virtues and emotional distress; and self-exertion to self and parents positively predict learning virtues but negatively predicts emotional distress.

## Materials and Methods

### Participants and Procedures

Convenience sampling was used to collect data. A total of 317 college students from seven public universities in Taiwan were recruited for the study. Students in Taiwan are admitted to college mainly based on their performance on academic subjects on the competitive college entrance examination. As public universities in Taiwan are generally considered more prestigious, they are more likely to recruit students with higher levels of academic performance. Such students are likely to take academic work seriously. They usually take around 3–6 required courses in their majors each semester. Participants in our study were recruited from four courses offered by these public universities. A questionnaire was administered in class immediately after the release of mid-term grades to gather their immediate recollections of a course with less satisfactory grade compared with other courses. It was assumed that the participants would have fresh memories of and responses to unsatisfactory grades in these courses. The grade distribution of 288 participants was as follows: 48 participants below 30 points, 127 participants between 30 and 59 points, 80 participants between 60 and 69 points, 29 participants between 70 and 79 points, 4 participants between 80 and 89 points, and none with 90–100 points, indicating that the majority of participants did choose courses with relatively unsatisfactory grades. Participants with missing values on the variables were deleted from the analysis. The valid sample size was 288, with 181 females (*M* = 21.90, *SD* = 3.75) and 91 males (*M* = 23.90, *SD* = 6.55).

### Instrument

All participants were asked to read and answer a questionnaire after they gave informed consents. The questionnaire included a recollection of failure on mid-term exams and 18 response items regarding the experience of failure. These items were divided into the following 5 subscales. A Likert scale ranging from 1 (strongly disagree) to 6 (strongly agree) was used.

#### Self-Exertion_personal (SE-P)

Self-exertion_personal, based on the aforementioned definition, meant fulfillment of one’s responsibility and duty to oneself. It consisted of two items: *“I have fulfilled my responsibility”* and *“I have exerted the utmost effort to fulfill my duty.”* Exploratory factor analysis revealed that a single factor accounted for 91.62% of the variance (Cronbach’s α = 0.91). The average of the two items indicated the participant’s level of duty fulfillment to him- or herself.

#### Self-Exertion_filial (SE-F)

Self-exertion_filial, also based on the definition mentioned above, meant fulfillment of one’s responsibility and duty to one’s parents. It consisted of two items: *“I have fulfilled my obligation to my parents” and “I don’t feel indebted to my parents because I have done my duty.”* Exploratory factor analysis revealed that a single factor accounted for 74.24% of the variance (Cronbach’s α = 0.65). The average of the two items indicated the participant’s level of duty fulfillment to his or her parents.

#### Effort

Effort was defined as intensity in time or energy used to pursue academic achievement and the persistence in that endeavor before the exam. Cooman et al. (2009) developed a work effort scale (WESC) with a three-factor structure, including direction (the behavior a person chooses to perform), intensity (how hard one works to perform the behavior), and persistence (how hard a person keeps trying to perform the behavior successfully). Five items were modified from the WESC to measure the level of effort. The five items were: “*I study hard for good grades”* (direction),“*I spent plenty of time on this subject/activity,” “I put a lot of energy into this subject/activity”* (intensity), *“I tried my best to figure out what’s difficult to understand,”* and *“I don’t give up easily in the face of difficulty on tests”* (persistence). Exploratory factor analysis revealed that the five items represented a single factor, which accounted for 58.56% of the variance (Cronbach’s α = 0.86). The average of the five items indicated the participants’ degrees of effort.

#### Learning Virtues

Learning virtues were defined as positive traits/qualities related to academic learning that are deemed to be morally good. Li (2012) proposed attributes of learning virtues, including earnestness, diligence, concentration, perseverance, endurance of hardship, and steadfastness. Six items were developed as follows: *I think I am a student with (1) commitment (2) diligence (3) perseverance (4) devotion (5) steadfastness (6) a good learning attitude.* Exploratory factor analysis revealed that the six items represented a single factor, which accounted for 78.28% of the variance (Cronbach’s α = 0.93). The average of the six items indicated the participants’ perceptions of their own learning virtues.

#### Emotional Distress

Emotional distress was defined as the level of negative affect derived from failure. Three items were adopted from previous research (Fwu et al., 2016b), including: *After I got my grade on the exam, I felt (1) upset (2) disappointed (3) depressed about my performance.* Exploratory factor analysis revealed that the three items represented a single factor, which accounted for 84.61% of the variance (Cronbach’s α = 0.91). The average of the three items indicated the participant’s level of negative emotions.

## Results

### Descriptive Statistics and Correlations

**Table 1** presents descriptive and correlational statistics for the variables of self-exertion_personal, self-exertion_filial, effort, learning virtues, and emotional distress.

**Table 1:** Descriptive statistics and inter-variable correlations (*N* = 288).

Variable	*M(SD)*	Correlation coefficient				
		(1)	(2)	(3)	(4)	(5)
(1) Self-exertion_personal	3.52 (1.33)	-				
(2) Self-exertion_filial	3.34 (1.20)	0.63^∗∗^	-			
(3) Effort	3.95 (1.13)	0.70^∗∗^	0.42^∗∗^	-		
(4) Learning virtues	3.91 (1.09)	0.52^∗∗^	0.35^∗∗^	0.52^∗∗^	-	
(5) Emotional distress	4.05 (1.25)	0.20^∗^	0.36	0.34^∗∗^	0.15^∗^	-

*^∗^p < 0.05, ^∗∗^ p < 0.01.*

### Test of Path Model

**FIGURE 2:**
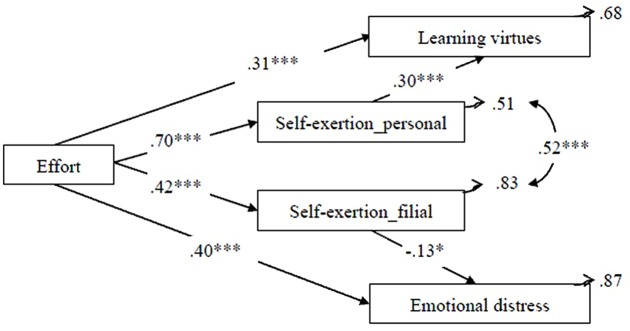
**The model of self-exertion as mediators between effort and learning virtues/emotional distress.** Only significant paths are included in the figure. Standardized coefficients are reported. ^∗∗∗^*p* < 0.001, ^∗^*p* < 0.05. Chi-square of χ^2^[3] = 0.299, *p* = 0.826, CFI = 1, AGFI = 0.994, RMSEA = 0.000, SRMR = 0.0095, GFI = 0.999.

Structural equation modeling was used to test our path model among effort, negative emotion, self-exertion, and learning virtues (see **Figure 2**). The results showed that the model fit the empirical data well [Chi-square of χ^2^[3] = 0.299, *p* = 0.826, CFI = 1, AGFI = 0.994, RMSEA = 0.000, SRMR = 0.0095, GFI = 0.999]. The model is presented in **Table 2**, with parameter estimates. Standardized parameter estimates are illustrated in **Figure 2**. Examination of these paths indicated six sets of relations. First, effort was positively correlated with self-exertion_personal (β = 0.697, *p* < 0.001), self-exertion_filial (β = 0.418, *p* < 0.001), and learning virtues (β = 0.310, *p* < 0.001). Second, effort was positively related to emotional distress (β = 0.396, *p* < 0.001). Third, self-exertion_personal was positively related to learning virtues (β = 0.301, *p* < 0.001). Finally, self-exertion_filial was positively related to learning virtues (β = -0.13, *p* < 0.05). We further applied bootstrapping to substantiate the mediation effect. The results showed that self-exertion_personal as a mediating variable between effort and learning virtues because the bootstrapped confidence interval of indirect effect did not include zero (95% C.I. = [0.109, 0.308]). Similarly, self-exertion_filial acted as a mediating variable between effort and emotional distress because the bootstrapped confidence interval of indirect effect did not include zero (95% C.I. = [-0.112, -0.008]).

**Table 2:** Parameter estimates and significant levels for the model.

	*Unstandardized coefficient*	*SE*	*Standardized coefficients*
Effort→Self-exertion_personal	0.821	0.050	0.697^∗∗∗^
Effort→Self-exertion_filial	0.446	0.057	0.418^∗∗∗^
Effort→Learning virtues	0.300	0.066	0.310^∗∗∗^
Effort→Emotional distress	0.439	0.067	0.396^∗∗∗^
Self-exertion_personal→Learning virtues	0.248	0.056	0.301^∗∗∗^
Self-exertion_filial→Emotional distress	-0.135	0.063	-0.130^∗^

*^∗^p < 0.05, ^∗∗∗^ p < 0.001.*

In summary, the model provided a good fit to the empirical data. All but two of the hypotheses were supported. Effort positively predicted SE-P (H-1) and SE-F (H-2), and effort also had a positive impact on learning virtues (H-3) and emotional distress (H-4). Furthermore, SE-P positively predicted learning virtues (H5-1), while SE-F (H6-2) negatively predicted emotional distress. The impacts of effort were mediated by self-exertion, in which SE-P was a source of learning virtues and SE-F was a buffer against negative affect. However, two hypotheses were not supported. SE-P could not predict emotional distress (H6-1), probably due to the relatively stronger prediction of SE-F than of SE-P. Similarly, SE-F could not predict learning virtues (H5-2), possibly because of the relatively greater effect of SE-P than of SE-F.

## Discussion

In summary, there appears to be a double-edged sword of effort, indicating that those who expend effort but fail tend to possess learning virtues but suffer from emotional distress. In addition, there is a shielding effect of self-exertion to parents, which reduces emotional distress from failure. The “sword and shield” effect is discussed in the following sections.

### Double-Edged Sword of Effort

In his studies on the effect of effort upon failure, Covington (1984, 2000, 2009) found that many students faced a “double-edged sword” between “making efforts to avoid teachers’ reproach” and “feeling incompetent owing to expending too much effort.” Behind this dilemma is the conflicting beliefs held by students and teachers. While students generally believe that trying too hard indicated a lack of ability (Nicholls, 1989), teachers tend to stress the work ethic and disapprove of students who did not expend enough effort on academic work (Hamilton et al., 1990; Weiner, 1993, 1994). These incompatible beliefs about ability vs. effort in academic achievement create the potential for inter-personal conflict between students and teachers. Covington’s notion of a ‘double-edged sword’ can apply both to failure in academic pursuits, such as math and science, and in non-academic activities, such as sports and the arts.

Unlike the inter-personal conflict between teachers and students in Covington’s study, previous studies conducted in Taiwan have shown that high school teachers and students all hold the same belief that effort is an act of virtue in fulfilling one’s duty (Fwu et al., 2014). No inter-personal conflict exists between the two groups. However, Taiwanese high school students also suffer from a double-edged sword of effort, an intra-personal conflict between a potential threat to one’s image and one’s emotional well-being (Fwu et al., 2016b). They are trapped in a dilemma between “feeling bad” (emotional distress) for exerting too much effort and “being bad” (negative image) for expending little effort. The trapping effect of effort is greater in the academic than in the non-academic domain. The present study corroborated these findings with Taiwanese college students and found that effortful college students were also faced with a double-edged sword, developing learning virtues but suffering emotional distress due to academic failure.

### Shielding Effect of Self-Exertion on Emotional Distress

Our study found that self-exertion to fulfill one’s duty to one’s parents has a shielding effect on emotional relief. Although the effect is relatively modest, it does reach statistical significance, implying such shielding effect exists and may open a window to explore this overlooked unique phenomenon. One possible explanation for this unique finding is that academic achievement is a culturally specific vertical goal for students in Taiwan. Parents expect their children to exert themselves to study hard and excel on the achievement pyramid. Although students who work hard but fail do not meet parental expectations of achieving academic excellence, they nevertheless tend to view themselves as partially meeting parental expectations and fulfilling their duty to their parents as filial children. Such positive appraisal of oneself may offset the negative feelings caused by one’s inability to achieve academic excellence. Moreover, when an individual believes that he has exerted himself, he will not feel he has failed his parents and may thus experience peace of mind and a clear conscience. It is clear that self-exertion to fulfill one’s role obligation to one’s parents acts as a shield to partially lessen one’s negative feelings.

This shielding effect of self-exertion to parents reflects the unique perspective of role obligation in Confucian culture (Hwang, 1999, 2012). In the Western tradition, which tends to hold an autonomous view of the self, individuals are socialized not to violate others’ rights nor to view others’ expectations as one’s own responsibility, which may have a negative effect of restricting one’s autonomy (Bedford and Hwang, 2003; Hwang, 2015). On the matter of academic learning, individuals have the right to choose whether they want to expend effort to pursue academic goals, and their parents’ will should not influence their choices. Academic learning is in essence is an individualized matter (Wigfield and Eccles, 1992, 2002; Eccles and Wigfield, 1995; Tao and Hong, 2000). In contrast, in the Confucian tradition, which stresses role ethics in the family (Ames, 2011), individuals are cultivated to play appropriate roles, such as those of parents and children, and to meet obligations inherent in the respective roles in the ethical relationship. For children, fulfilling the role of the filial child is a must, and conforming to parental expectations, an act of filial piety. As pursuit of academic achievement is often viewed as a vertical goal that is expected by their parents, children have little choice but to expend effort to pursue the goal. Thus, academic learning is not an individualized matter but a social obligation (Tao and Hong, 2000, 2014; Li, 2002, 2005; Tseng, 2004; Hau and Ho, 2010). When an individual exerts his utmost effort to study hard academically, he will feel that he has fulfilled his obligation to his parents. Such cognition of being filial and ethical through self-exertion to his parents may provide the individual with a sense of worth that may shield him from the psychological distress of failure.

### Limitations and Future Research

This study has several limitations. First, the data presented are correlational and thus do not imply causal directions. Relations among the variables are likely to be bidirectional. Conclusions concerning cause-and-effect should be drawn with caution. Second, our data were gathered from self-reports based on individuals’ intentions, rather than on actual behaviors, and on recollections of past experiences, rather than on responses to current situations. Caution should be exercised in making inferences about individuals’ actual behaviors. Third, each participant was asked to select one course grade that was less satisfactory when compared with other required courses. However, since everyone may hold different criteria for assessing level of satisfaction with one’s own grade, it is suggested that future research measure directly the subjective self-perception of one’s performance on a course to investigate the impact of one’s subjective perception on emotional distress. Fourth, since the degree of shielding effect was not high, but reach the level of statistically significant, it is advisable to increase the items measuring the constructs of self-exertion-personal and self-exertion-filial to substantiate the effect of self-exertion on emotional distress. In addition to the emotional effect, it would be interesting to further investigate the relationship between self-exertion and attributional patterns (cognitive), moral sentiment (affective), and future engagement (behavioral). Finally, self-exertion has an effect on learning virtues and emotional distress in academic failure. It would be worthwhile to further explore the degree to which self-exertion is also needed in situations of academic success and its effect on individual psychological processes.

## Author Contributions

All authors substantially contribute to the conception, analysis, and interpretation of data for the work; and revise it critically for important intellectual content; and finally approve the version to be published; and agree to be accountable for all aspects of the work in ensuring that questions related to the accuracy or integrity of any part of the work are appropriately investigated and resolved.

## Conflict of Interest Statement

The authors declare that the research was conducted in the absence of any commercial or financial relationships that could be construed as a potential conflict of interest.
